# Supplementary material for: Time-series transcriptome analysis identified differentially expressed genes in broiler chicken infected with mixed Eimeria species
Source: Front Genet. 2022 Aug 8;13:886781. doi: 10.3389/fgene.2022.886781 (PMC9393255; doi:10.3389/fgene.2022.886781)
Supplement: Supplementary file 2 [file DataSheet1.ZIP › 7dpi_KEGG.Gsea.1625115961950/KEGG_PARKINSONS_DISEASE.html]

Details for gene set KEGG\_PARKINSONS\_DISEASE[GSEA]

|  || Dataset | TMM\_7dpi\_gct\_format\_7dpi\_gct\_format.Class\_4dpi.cls #PC\_versus\_NC.Class\_4dpi.cls #PC\_versus\_NC\_repos |
| Phenotype | Class\_4dpi.cls#PC\_versus\_NC\_repos |
| Upregulated in class | 1 |
| GeneSet | KEGG\_PARKINSONS\_DISEASE |
| Enrichment Score (ES) | 0.693416 |
| Normalized Enrichment Score (NES) | 3.363091 |
| Nominal p-value | 0.0 |
| FDR q-value | 0.0 |
| FWER p-Value | 0.0 |
Table: GSEA Results Summary

  

Fig 1: Enrichment plot: KEGG\_PARKINSONS\_DISEASE      
 Profile of the Running ES Score & Positions of GeneSet Members on the Rank Ordered List

  

| SYMBOL | TITLE | RANK IN GENE LIST | RANK METRIC SCORE | RUNNING ES | CORE ENRICHMENT || 1 | UBE2L6 | na | 141 | 1.154 | 0.0192 | Yes |
| 2 | SLC25A5 | na | 276 | 0.959 | 0.0337 | Yes |
| 3 | COX5A | na | 300 | 0.942 | 0.0571 | Yes |
| 4 | NDUFA2 | na | 303 | 0.935 | 0.0821 | Yes |
| 5 | NDUFB6 | na | 307 | 0.933 | 0.1069 | Yes |
| 6 | NDUFS5 | na | 308 | 0.932 | 0.1319 | Yes |
| 7 | NDUFB7 | na | 323 | 0.915 | 0.1553 | Yes |
| 8 | NDUFB2 | na | 348 | 0.885 | 0.1771 | Yes |
| 9 | NDUFAB1 | na | 356 | 0.876 | 0.2000 | Yes |
| 10 | NDUFA7 | na | 371 | 0.857 | 0.2219 | Yes |
| 11 | NDUFC2 | na | 394 | 0.834 | 0.2424 | Yes |
| 12 | UQCR10 | na | 444 | 0.785 | 0.2594 | Yes |
| 13 | CYCS | na | 475 | 0.763 | 0.2774 | Yes |
| 14 | COX6A1 | na | 480 | 0.760 | 0.2975 | Yes |
| 15 | UQCR11 | na | 485 | 0.754 | 0.3174 | Yes |
| 16 | UQCRB | na | 487 | 0.753 | 0.3376 | Yes |
| 17 | COX7A2 | na | 499 | 0.743 | 0.3566 | Yes |
| 18 | NDUFS3 | na | 623 | 0.675 | 0.3644 | Yes |
| 19 | NDUFB3 | na | 656 | 0.657 | 0.3794 | Yes |
| 20 | NDUFS8 | na | 661 | 0.653 | 0.3966 | Yes |
| 21 | NDUFS6 | na | 686 | 0.642 | 0.4118 | Yes |
| 22 | SDHD | na | 708 | 0.632 | 0.4271 | Yes |
| 23 | SLC18A2 | na | 715 | 0.628 | 0.4434 | Yes |
| 24 | NDUFA1 | na | 742 | 0.616 | 0.4578 | Yes |
| 25 | UQCRHL | na | 788 | 0.594 | 0.4700 | Yes |
| 26 | NDUFA5 | na | 794 | 0.593 | 0.4855 | Yes |
| 27 | SDHB | na | 840 | 0.575 | 0.4972 | Yes |
| 28 | NDUFB5 | na | 902 | 0.554 | 0.5070 | Yes |
| 29 | COX6C | na | 930 | 0.546 | 0.5194 | Yes |
| 30 | NDUFV3 | na | 988 | 0.527 | 0.5288 | Yes |
| 31 | NDUFB4 | na | 1015 | 0.517 | 0.5405 | Yes |
| 32 | COX7B | na | 1055 | 0.501 | 0.5506 | Yes |
| 33 | NDUFA6 | na | 1079 | 0.492 | 0.5619 | Yes |
| 34 | NDUFA8 | na | 1080 | 0.492 | 0.5752 | Yes |
| 35 | UQCRQ | na | 1103 | 0.484 | 0.5863 | Yes |
| 36 | NDUFS2 | na | 1124 | 0.477 | 0.5975 | Yes |
| 37 | COX4I1 | na | 1125 | 0.476 | 0.6103 | Yes |
| 38 | SDHC | na | 1135 | 0.473 | 0.6222 | Yes |
| 39 | NDUFB10 | na | 1215 | 0.450 | 0.6277 | Yes |
| 40 | VDAC2 | na | 1244 | 0.443 | 0.6372 | Yes |
| 41 | UQCRFS1 | na | 1263 | 0.437 | 0.6475 | Yes |
| 42 | ATP5PB | na | 1267 | 0.436 | 0.6589 | Yes |
| 43 | NDUFV1 | na | 1307 | 0.428 | 0.6672 | Yes |
| 44 | NDUFB8 | na | 1330 | 0.423 | 0.6767 | Yes |
| 45 | NDUFS7 | na | 1371 | 0.412 | 0.6844 | Yes |
| 46 | NDUFV2 | na | 1395 | 0.408 | 0.6934 | Yes |
| 47 | UQCRC2 | na | 1700 | 0.332 | 0.6769 | No |
| 48 | COX7A2L | na | 1761 | 0.316 | 0.6803 | No |
| 49 | UBE2L3 | na | 1809 | 0.305 | 0.6846 | No |
| 50 | NDUFB9 | na | 1842 | 0.299 | 0.6899 | No |
| 51 | NDUFB1 | na | 1995 | 0.272 | 0.6845 | No |
| 52 | CASP9 | na | 2022 | 0.268 | 0.6895 | No |
| 53 | HTRA2 | na | 2060 | 0.261 | 0.6934 | No |
| 54 | UQCRC1 | na | 2326 | 0.217 | 0.6770 | No |
| 55 | NDUFA4 | na | 2726 | 0.160 | 0.6479 | No |
| 56 | UBB | na | 2862 | 0.144 | 0.6405 | No |
| 57 | NDUFA9 | na | 2879 | 0.141 | 0.6429 | No |
| 58 | NDUFA10 | na | 3412 | 0.073 | 0.6003 | No |
| 59 | SDHA | na | 3488 | 0.064 | 0.5957 | No |
| 60 | SLC18A1 | na | 3610 | 0.051 | 0.5869 | No |
| 61 | NDUFS1 | na | 3763 | 0.033 | 0.5751 | No |
| 62 | PARK7 | na | 3768 | 0.032 | 0.5756 | No |
| 63 | UBE2G1 | na | 3860 | 0.021 | 0.5686 | No |
| 64 | PPID | na | 4134 | -0.010 | 0.5459 | No |
| 65 | UBE2G2 | na | 4360 | -0.036 | 0.5280 | No |
| 66 | NDUFS4 | na | 4402 | -0.040 | 0.5257 | No |
| 67 | COX7C | na | 4881 | -0.090 | 0.4881 | No |
| 68 | UBE2J1 | na | 4897 | -0.092 | 0.4893 | No |
| 69 | SNCA | na | 5117 | -0.114 | 0.4740 | No |
| 70 | SLC25A4 | na | 5331 | -0.133 | 0.4597 | No |
| 71 | VDAC3 | na | 5532 | -0.152 | 0.4470 | No |
| 72 | SLC25A6 | na | 6201 | -0.225 | 0.3971 | No |
| 73 | UBE2J2 | na | 6415 | -0.245 | 0.3858 | No |
| 74 | CASP3 | na | 6448 | -0.248 | 0.3898 | No |
| 75 | UCHL1 | na | 8498 | -0.483 | 0.2311 | No |
| 76 | LRRK2 | na | 8529 | -0.487 | 0.2416 | No |
| 77 | APAF1 | na | 8687 | -0.512 | 0.2422 | No |
| 78 | SNCAIP | na | 9443 | -0.623 | 0.1957 | No |
| 79 | VDAC1 | na | 10060 | -0.721 | 0.1634 | No |
Table: GSEA details [plain text format]

  

Fig 2: KEGG\_PARKINSONS\_DISEASE      
 Blue-Pink O' Gram in the Space of the Analyzed GeneSet

  

Fig 3: KEGG\_PARKINSONS\_DISEASE: Random ES distribution      
 Gene set null distribution of ES for **KEGG\_PARKINSONS\_DISEASE**
